# Supplementary material for: What does it cost to expand two-way texting for post-operative follow-up? A cost analysis in routine voluntary medical male circumcision settings in South Africa
Source: medRxiv. 2024 Nov 28:2024.11.26.24317997. Preprint. [Version 1] doi: 10.1101/2024.11.26.24317997 (PMC11623716; doi:10.1101/2024.11.26.24317997)
Supplement: Supplement 1 [file NIHPP2024.11.26.24317997v1-supplement-1.pdf]

medRxiv preprint doi: https://doi.org/10.1101/2024.11.26.24317997; this version posted November 28, 2024. The copyright holder for this preprint (which was not certified by peer review) is the author/funder, who has granted medRxiv a license to display the preprint in perpetuity.

It is made available under a CC-BY 4.0 International license .

Supporting information

S1 Table. Costing tool for 2wT implementation. Modifiable Excel spreadsheet for 2wT costing during stepped wedge expansion study and for additional scenario analysis.
